# Auditory Inspired Convolutional Neural Networks for Ship Type Classification with Raw Hydrophone Data

**DOI:** 10.3390/e20120990

**Published:** 2018-12-19

**Authors:** Sheng Shen, Honghui Yang, Junhao Li, Guanghui Xu, Meiping Sheng

**Affiliations:** School of Marine Science and Technology, Northwestern Polytechnical University, Xi’an 710072, China

**Keywords:** convolutional neural network, deep learning, auditory, ship radiated noise, hydrophone

## Abstract

Detecting and classifying ships based on radiated noise provide practical guidelines for the reduction of underwater noise footprint of shipping. In this paper, the detection and classification are implemented by auditory inspired convolutional neural networks trained from raw underwater acoustic signal. The proposed model includes three parts. The first part is performed by a multi-scale 1D time convolutional layer initialized by auditory filter banks. Signals are decomposed into frequency components by convolution operation. In the second part, the decomposed signals are converted into frequency domain by permute layer and energy pooling layer to form frequency distribution in auditory cortex. Then, 2D frequency convolutional layers are applied to discover spectro-temporal patterns, as well as preserve locality and reduce spectral variations in ship noise. In the third part, the whole model is optimized with an objective function of classification to obtain appropriate auditory filters and feature representations that are correlative with ship categories. The optimization reflects the plasticity of auditory system. Experiments on five ship types and background noise show that the proposed approach achieved an overall classification accuracy of 79.2%, which improved by 6% compared to conventional approaches. Auditory filter banks were adaptive in shape to improve accuracy of classification.

## 1. Introduction

Ship radiated noise is one of the main sources of ocean ambient noise, especially in coastal waters. Hydrophones provide real-time acoustic measurement to monitor underwater noise in chosen areas. However, automatic detection and classification of ship radiated noise signals are still quite difficult at present because of multiple operating conditions of ships and complexity of sound propagation in shallow water. Various signal processing strategies have been applied to address these problems. Most of the efforts focus on extracting features and developing nonlinear classifiers.

Extracting appropriate ship radiated noise features has been an active area of research for many years. Hand designed features always describe ship radiated noise in terms of waveform, spectral and cepstral characteristics. Zero-crossing features and peak-to-peak amplitude features [1,2] were presented to describe rotation of propeller, but their performances were greatly reduced in noisy shallow seas. Features based on wavelet packet [3] were extracted, but they were difficult to determine decomposition series of wavelet. In addition, multiscale entropy method [4] was proposed to detect and recognize ship targets. Spectral [5] features and cepstral coefficients features [5,6] were extracted. However, these methods always suffer a lot from limited priori knowledge of datasets. Auditory models have been shown to work well for a variety of audio processing tasks. As for ship classification, auditory features based on dissimilarity evaluation was proposed [7]. Mel-frequency cepstral coefficients (MFCC) were applied to describe ship radiated noise [8]. However, the widely used auditory filter bank models of cochlea assume that, for a given center frequency, there is a fixed filter bandwidth, but this property is not well matched by reverse correlation data in auditory experiments [9].

Designing appropriate classifiers given hand designed features has been another active area of research. Multiple Support Vector Machine (SVM) classifiers [10] were integrated to improve classification accuracy and robustness, but it was always inefficient. Neural classifiers [11] based on a feed-forward neural network were studied. Four conventional neural networks and average power spectral density features [12] were used to classify underwater signals. Probabilistic linear discriminant analysis, i-vectors and neural networks [13] motivated by speech related technologies were performed to individual ship detection. Neural networks and i-vector features [14] were used to detect ship presence and classify ship types, and the authors also discussed the influence of a set of data preprocessing technologies on recognition results. Class-modular multi-layer perceptron and spectral features [15] were used to classify ships. All of them used Fourier transform based features and shallow neural networks. Generally, classifier design and feature extraction were separate from each other. This has a drawback that the designed features may not be best for the classification task. As for classification models based on auditory features, auditory filter banks designed from perceptual evidence always focus on the properties of signal description rather than the classification purpose [16].

Deep learning has made it possible for modeling original signal as well as predicting targets in a whole model, to which the human auditory system is thought to be adapted. Kamal [17] used a deep belief network and Cao [18] used a sparse deep auto encoder. A competitive learning mechanism based deep learning model [19] and its compression algorithm [20] were proposed to increase clustering performance. All of them trained deep neural networks on discrete Fourier transform (DFT) magnitude features. These works demonstrated that deep neural networks can be used to learn a better representation using lower level frequency magnitude based features. However, they were unable to make use of information found in time structure.

In this paper, an auditory inspired convolutional neural network is proposed to simulate the processing procedure of human auditory system for ship type classification. The network is trained from raw hydrophone data in an end-to-end manner to simulate auditory pathway, rather than designing features and classifiers separately. In the proposed model, the shallow layer performs signal decomposition by convolution operation to simulate cochlea, and deep layers perform class-based discrimination to simulate auditory cortex. The learned filters in shallow layer and learned features in deep layers are subject to classification tasks on the basis of matching human auditory systems.

This paper is organized as follows. Section 2 gives an overview of the auditory inspired convolutional neural network for ship type classification. Section 3 and Section 4 describe individual processing chain components, which includes auditory filter banks learning for ship radiated noise modeling, deep features’ learning and ship targets’ classification. Experimental data description, experimental setup and results are presented and discussed in Section 5. An overall discussion and directions for future work are concluded in Section 6.

## 2. Architecture of Auditory Inspired Convolutional Neural Network for Ship Type Classification

The human auditory system has a remarkable ability to deal with the task of ship radiated noise classification [21,22]. It is of practical significance to establish the human auditory system mathematically. The human auditory system includes two basic regions of stimulus processing. The first region is the peripheral region [23]. In this region, the incoming acoustic signal is transmitted mechanically to the inner ear, and it is decomposed into frequency components at the cochlea. The second region of the system gets the neural signal to form auditory perception in the auditory cortex; therefore, the listener could discriminate between different sounds. The nature of the auditory model is to transform a raw acoustical signal into representations that are useful for auditory tasks [9].

**Figure 1:**
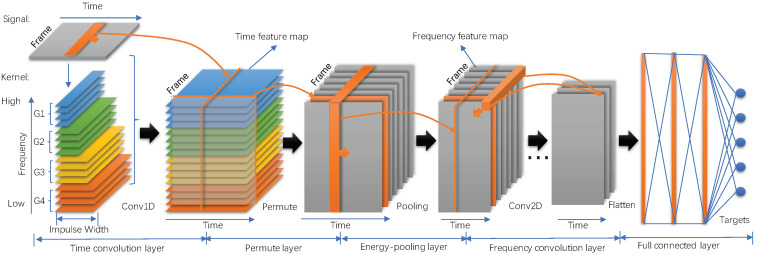
Auditory inspired convolutional neural network structure. In the time convolutional layer, four colors represent four groups of auditory filters with different center frequencies and impulse widths. In the permute layer and energy-pooling layer, decomposed signals are converted to frequency feature maps, each of which correspond to a frame. In frequency convolutional layers, convolution operations are implemented in both time and frequency axis. At the end of the network, several full connected layers and target layers are used to predict targets.

In this paper, the two regions of the auditory system are simulated for ship type classification in a whole model named the auditory inspired convolutional neural network. The structure of the proposed model is shown in Figure 1. The proposed model includes three parts: the first part is inspired by the cochlea and takes raw underwater acoustic data as an input. This part is performed by a 1D time convolutional layer. The convolutional kernels are initialized by Gammatone filters based on the research foundation of human cochlea. A collection of decomposed intrinsic modes can be generated in the output of this layer. The second part is inspired by the auditory cortex and takes the output of the time convolutional layer as input signals. This part includes a permute layer, energy-pooling layer, 2D frequency convolutional layer and full connected layer. The permute layer and energy-pooling layer could convert the decomposed signal into a frequency domain. The 2D frequency convolutional layers are applied to preserve locality and reduce spectral variations in ship noise. In the third part, the whole model is optimized with an objective function of ship type classification. A more general way to express the process is: the time convolutional layer yields different simple intrinsic modes of ship noise that help the feature learning at deep layers and help ship targets’ classification at the output layer. At the same time, the Gammatone filters and features are optimization by CNN to obtain appropriate representations that are correlative with ship categories.

## 3. Learned Auditory Filter Banks for Ship Radiated Noise Modeling

The response properties of cochlea have been studied extensively. In cochlea, signals are encoded with a set of kernels. The kernels can be viewed as an array of over-lapping band pass auditory filters that occur along basilar membrane. These filters’ center frequencies increase from the apex to the base of the cochlea. In addition, their bandwidths are much narrower at lower frequencies [24,25]. This property is appropriate for describing ship radiated noise, since the energy of ship radiated noise is mainly concentrated in lower frequencies.

### 3.1. Auditory Filter Banks and Time Convolutional Layer

One solution of mathematical approximations of cochlea filter banks is Gammatone kernel functions (Gamma-modulated sinusoids) [26], which are linear filters described by impulse responses. The Gammatone impulse response is given by:

(1) $$g\left(t\right)=at^{n-1}e^{-2\pi bt}\cos\left(2\pi ft+\phi \right),$$

where *f* is center frequency in Hz, ϕ is phase of the carrier in radians, *a* is amplitude, *n* is filter’s order, *b* is bandwidth in Hz, and *t* is time in seconds. Center frequency and bandwidth are set according to an equivalent rectangular bandwidth (ERB) filter bank cochlea model, which is approximated by the following equation [27]:

(2) ERB(f)=24.7(4.37f/1000+1),

(3) b=1.019×ERB(f).

**Figure 2:**
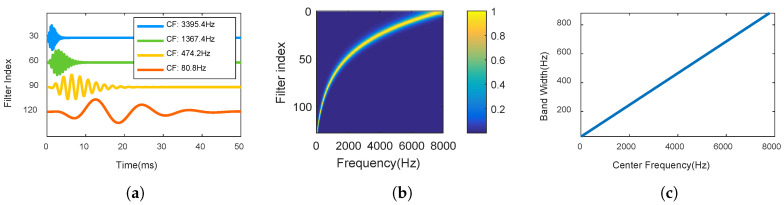
Gammatone auditory filters. (**a**) four time domain filters with different center frequencies (CF); (**b**) frequency magnitude responses of all 128 filters; (**c**) relationship between center frequencies and bandwidths.

In this paper, 128 Gammatone filters with center frequencies range from 20 Hz to 8000 Hz are generated. Four Gammatone filters are shown in Figure 2a. Figure 2b shows magnitude responses of Gammatone filter banks, and Figure 2c shows the relationship between center frequencies and bandwidths.

However, Gammatone filters need to be optimized for the following reasons: (1) There is a fixed bandwidth for a given center frequency. This assumption is not matched by auditory reverse correlation data, which show a range of bandwidths at any given frequency [9]; (2) ERB filter bank cochlea model provides linear filters, which doesn’t account for nonlinear aspects of the auditory system [27]; (3) Auditory filter banks designed from perceptual evidence always focus on the properties of signal description rather than the classification purpose [16].

The first layer in the proposed CNN architecture is a time convolutional layer over a raw time domain waveform. CNN is a kind of artificial neural network which performs a series of convolutions over input signals. We use a physiologically derived set of Gammatone filters to initial this layer for sound representation. The output of each filter is mathematically expressed as convolution of the input with impulse response. Then, convolutional kernels can be interpreted as representing a population of auditory nerve spikes. As shown in Equation (4), waveform *x* is convolved with trainable Gammatone kernel $k_{j}$ and put through activation function *f* to form the output feature map $y_{j}$. Each output feature map is given an additive bias $b_{j}$. The time convolutional operation is only on the time axis. There is one output for each kernel and the dimensionality of each output is identical to the input:

(4) $$y_{j}=f\left(x*k_{j}+b_{j}\right).$$

To optimize the kernel functions, a gradient-based algorithm is derived to update them along with parameters in deep layers. The optimized convolutional kernels can be viewed as a set of band-pass finite impulse response filters which correspond to different locations of the basilar membrane. The relationship between center frequencies and bandwidths is optimized to match the classification task.

### 3.2. Multi-Scale Convolutional Kernels

**Figure 3:**
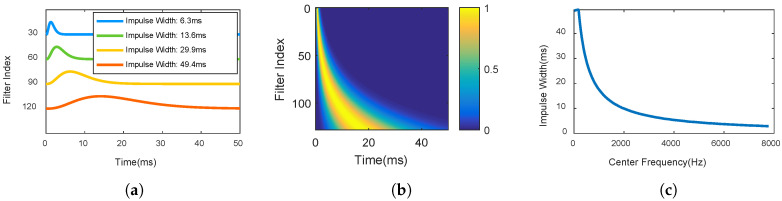
Gamma envelopes of Gammatone filters. (**a**) gamma envelops of four filters; (**b**) magnitude of gamma envelopes of all 128 filters; (**c**) relationship between impulse widths and center frequencies.

Gammatone filters with similar center frequencies are more correlative with each other and they always have similar impulse widths. Gamma envelopes of Gammatone filters are shown in Figure 3a,b. The relationship between impulse widths and center frequencies is shown in Figure 3c. The impulse widths range from 50 to 800 points for 16 kHz sampling frequency. The impulse widths get wider for lower frequencies.

As suggested by Arora [28], in a layer-by-layer construction, correlation statistics of each layer should be analyzed by clustering it into groups of units with high correlation. In this paper, filters with similar impulse widths are clustered in one group. The grouping of filters is performed by quartering, with each of the groups having the same number of filters. The width of the four groups are set as 100, 200, 400, and 800 points, respectively. In each group, we create multiple shifted copies of each filter’s impulse response. Another parameter to be selected for the time convolutional layer is the number of kernel functions. Filter banks with more than 16 Gammatone kernels have more than necessary, but increasing the number allows greater spectral precision [29]. We used a set of 32 kernel functions in each group.

The multi-scale convolutional kernels have several advantages: first, convolutional kernels with varying lengths could cover multi-scale reception field to provide a better description of sounds. Second, correlation statistics of signal components can be analyzed by filter bank groups. Third, fewer parameters in multi-scale kernels can prevent overfitting and save on computing resources, especially for filters with narrower impulse width.

## 4. Auditory Cortex Inspired Discriminative Learning for Ship Type Classification

In an auditory system, cochlear nerve fibers at the periphery are narrowly tuned in frequency [30]. In the proposed model, a time convolutional layer yields representations that correspond to different frequencies. An auditory cortex is involved in tasks such as segregating and identifying auditory “objects”. Neurons in the primary cortex have shown to be sensitive to specific spectro temporal patterns in sounds [30], and they are likely to reflect the fact that the cochlea is arranged according to frequency. Inspired by this property, we proposed the permute layer, energy-pooling layer and frequency convolutional layer.

### 4.1. Permute Layer and Energy-Pooling Layer

After the time convolutional layer, the rest of the network is constructed by first converting each output into a time-frequency distribution. This is one way of describing the information our brains get from our ears. Assuming that the output of time convolutional layer has a dimension of l×n×m, where *l* is frame length, *n* and *m* represent the number of frames and time feature maps, respectively. This output is permuted into dimension of l×m×n in the permute layer, thus we get *n* output feature maps, each of which correspond to a frame and has a dimension of l×m. Then, each output feature map is pooled over the entire frame length by computing root-mean-square energy in the energy-pooling layer, so that the energy of each signal component is summed up within regular time bins. Layer normalization is applied to normalize the energy sequences.

**Figure 4:**
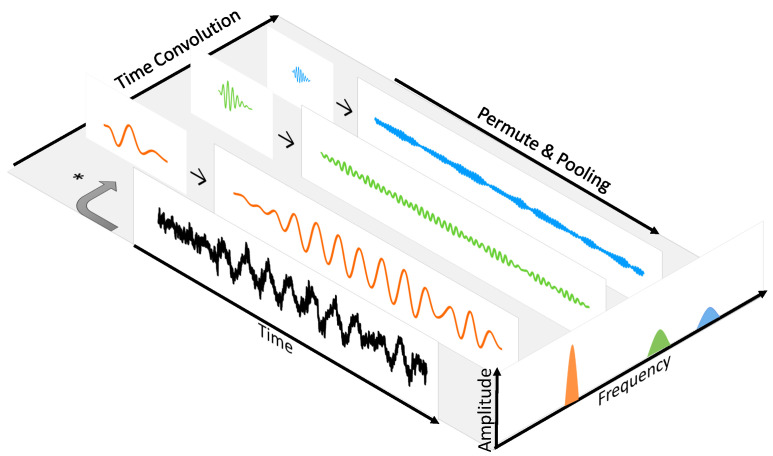
The length of the passenger ship is 139 m. The recording segment is 250 ms. During the recording period, the ship is 1.95 km away from the hydrophone and its navigational speed is 18.4 kn. Its radiated noise is convolved with each of three Gammatone filters. Their center frequencies are 49 Hz (orange line), 194 Hz (green line) and 432 Hz (blue line). Energy of each component is summed up to convert to a frequency domain.

Figure 4 illustrates the decomposition of a time domain waveform and time-frequency conversion by using underwater noise radiated from a passenger ship. The filters’ outputs show that the waveform is decomposed into corresponding frequency components. The bottom right part of Figure 4 shows that each component is converted into a frequency domain.

### 4.2. Frequency Convolutional Layer and Target Layer

Neurons in the primary auditory cortex have complex patterns of sound-feature selectivity. These patterns indicate sensitivity to stimulus edges in frequency or in time, stimulus transitions in frequency or intensity, and feature conjunctions [31]. Thus, in the proposed model, several 2D frequency convolutional layers are applied to discover time-frequency edge of the ship radiated noise based on the output of pooling layer. Convolution operations are performed in both time and frequency axis. The dimension of convolutional kernel is 3×3. The creation of frequency convolutional layer matched to the processing characteristics of auditory cortical neurons. These layers could also preserve locality and reduce spectral variations of line spectrum in ship radiated noise.

The output from the last frequency convolutional layer is flattened to form the input of a full connected layer. To obtain a probability over every ship type for each sample, the end of the network is a softmax target layer and the loss function is a categorical cross entropy. Output y is computed by applying the softmax function to the weighted sums of the hidden layer activation s. The *i*th output $y_{i}$ is:

(5) $$y_{i}=\frac{e^{s_{i}}}{\sum_{c}^{nclass}e^{s_{c}}}.$$

The cross entropy loss function *E* for multi-class output is:

(6) $$E=-\sum_{i}^{nclass}t_{i}log(y_{i}),$$

where t is the target vector. Parameters of both time convolutional layer, frequency convolutional layers and full connected layers are optimized jointly with the softmax target layer. Both auditory filter banks and feature representations are optimized correlative with ship category by an optimization algorithm that reflects the plasticity of an auditory system.

## 5. Experiments and Discussion

### 5.1. Experimental Dataset

**Figure 5:**
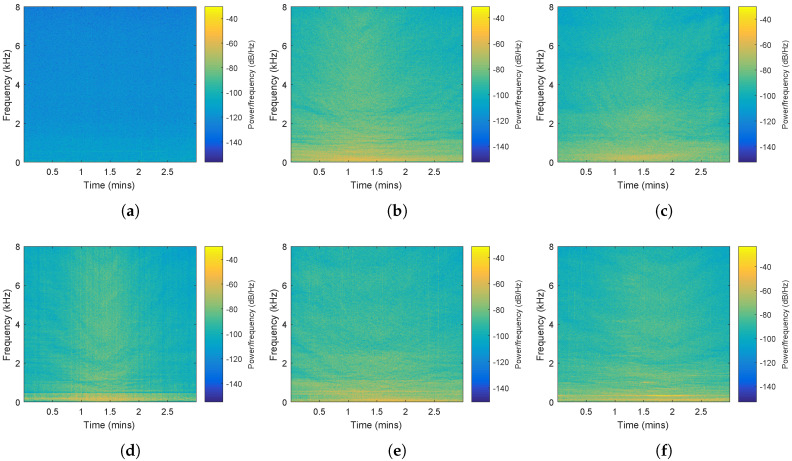
Spectrogram of hydrophone signal for each category. (**a**) background noise; (**b**) cargo; (**c**) passenger ship; (**d**) pleasure craft; (**e**) tanker; (**f**) tug.

Our experiments were performed on a 64-h measured ship radiated noise acquired by Ocean Networks Canada observatory. Acoustic data were measured using an Ocean Sonics icListen AF hydrophone placed at Latitude 49.00811°, Longitude −123.33906° and 144 m below sea level. Sampling frequency of the signal was 32 kHz and it was down sampled to 16 kHz in our experiments. The acquired acoustic data were combined with Automatic Identification System data. Ships during normal operating conditions presented in an area of 2 km radius of the hydrophone deployment site were recorded. To minimize noise generated by other ships, there were no other ships presented in a 3 km radius of the hydrophone deployment site for each recording. Ship categories of interest are Cargo, Passenger ship, Pleasure craft, Tanker and Tug. Classification experiments were performed on the five ship categories and background noise. Spectrograms of signals for these classes are shown in Figure 5.

The acquired original dataset has 474 recordings. Each recording can be sliced into serval segments to make up the input of a neural network. The length of segments used for classification can be adjusted according to the acquired signal; then, the input layer of the network should be adjusted accordingly. Every segment was classified independently. The classification results obtained on 3 s segments were more stable and accurate than classified with shorter segments. This may be because burst noise in the acquired signal has a greater negative impact on the recognition of short segments. For a given network structure, longer segments result in greater space complexity. For the limitation of memory capacity, a training network with longer segments requires a smaller batch size and even causes out-of-memory errors. Considering the computational ability and classification accuracy, the experiments in this paper were performed on segments of 3 s duration. Thus, the dataset consists of 76,918 segments. For each category, about 10,000 segments were used for training and 2500 segments were used for testing. In order to simulate real application situation, segments in one recording can’t be split into a training dataset and test dataset. Each signal was divided into short frames of 256 ms, so each sample is a 4096×12 data matrix.

### 5.2. Classification Experiments

The classification performance of the proposed method was compared to same structure CNNs with a randomly initialed time convolutional layer and untrainable Gammatone initialed time convolutional layer. The proposed method was also compared to CNNs trained on hand designed features. These hand designed features included waveform features, wavelet features, MFCC, Mel-frequency features, nonlinear auditory features, spectral and cepstral features. The two pass split window (TPSW) [32] is applied subsequently after short-time fast Fourier transform for the enhancement of the signal-to-noise ratio (SNR). The TPSW filtering scheme provides a mechanism for obtaining smooth local-mean estimates of the signal. Mel-frequency features were extracted by calculating log Mel-frequency magnitude. Nonlinear auditory filters [33] with 128 channels were utilized to extract nonlinear auditory features. First, 512-cepstral coefficients were extracted as cepstral features. Other features were described in our previous paper [20]. Each signal was windowed into 256 ms frames before extracting features. The extracted features on frames were stacked to create a feature vector. The tensorflow Python library runs on a NVIDIA GTX1080 graphics card (Santa Clara, CA, USA), which was used to perform the bulk of the computations. Table 1 shows the CNNs structure for classification experiments. Table 2 shows the hyper-parameters of the proposed model.

Table 3 shows the classification performance of different approaches. The proposed model achieved the highest accuracy of 79.2%. The baseline system, CNN trained on spectral features, had a classification accuracy of 73.2%. The proposed method gave 6% improvement in accuracy compared to the baseline. The accuracy of proposed model was apparently higher than CNNs trained on other hand designed features. CNN with randomly initialed time convolutional layer and untrainable Gammatone initialed time convolutional layer gave an accuracy of 60.8% and 75.3%, respectively. The results indicated that auditory filter banks together with a back propagation algorithm helped CNN to discover better features.

Table 4 shows the precision, recall and f1-score obtained from the confusion matrix of the proposed model. The background noise class had the highest recall value of 0.94, while the precision was only 0.73. This indicted that ships cannot be detected when they were far away from the hydrophone. The ship classes with the best results were Cargo and Passenger, with f1-score of 0.87 and 0.86, respectively. The poorest results were obtained for Tug, with precision of 0.73, recall of 0.54 and f1-score of 0.62. This may be because tugs have a similar mechanical system with other classes or some tugs were towing other ships during the recoding period.

**Figure 6:**
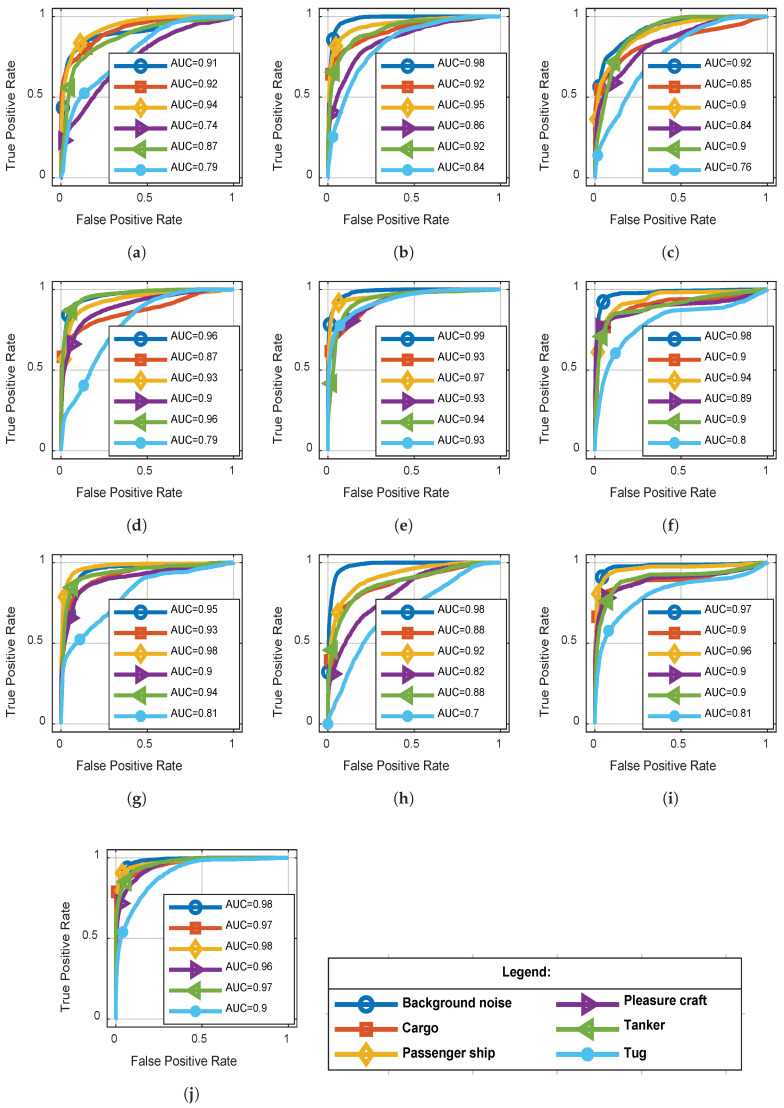
ROC curves of the classification results for all methods by assuming that one class was positive and other classes were negative. (**a**) waveform features; (**b**) wavelet features; (**c**) MFCC; (**d**) mel-frequency; (**e**) nonlinear auditory features; (**f**) spectral; (**g**) cepstral; (**h**) untrainable Gammatone initialed; (**i**) randomly initialed; (**j**) proposed method.

Receiver operating characteristic (ROC) curves were constructed by the output of a softmax layer obtained on test data, assuming that one class was positive and other classes were negative. Figure 6 shows the ROC curves and area under curve (AUC) values obtained by different approaches. Performances of the proposed model shown in Figure 6j were significantly better than other methods for almost all classes. The accuracies of background noise were always higher than other classes, which indicated that it was easier in detecting ships’ presence than classifying ship types.

### 5.3. Visualization and Analysis

#### 5.3.1. Visualization and Analysis of Learned Filters

**Figure 7:**
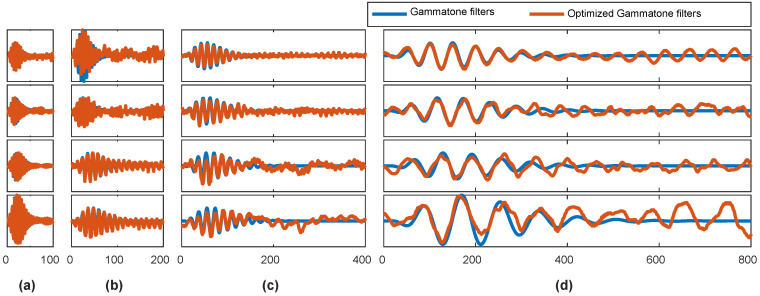
The comparison of optimized Gammatone kernels and conventional Gammatone kernels. (**a**) filters in group 1; (**b**) filters in group 2; (**c**) filters in group 3; (**d**) filters in group 4.

Initializing CNN weights by Gammatone filters, the CNN managed to optimize impulse responses during its training process. Figure 7 shows the optimized Gammatone kernels for ship radiated noise in the proposed model. The algorithm modified amplitude and impulse width of Gammatone filters, but temporal asymmetry and gradual decay of the envelope that match the physiological filtering properties of auditory nerves were reserved.

We can also compare population properties of optimized Gammatone filters with those of conventional Gammatone filters. To illustrate spectral properties of optimized filters, we zero-padded every time convolutional kernel $w_{i}$ to 800 entries, and then calculated the magnitude spectrum $W_{i}$:

(7) $$W_{i}=\left|\mathrm{DFT}\left(w_{i}\right)\right|,1\leq i\leq 128.$$

Center frequency $f_{c}^{i}$ was calculated as the position of the maximum magnitude:

(8) $$f_{c}^{i}=\mathrm{argmax}\left(W_{i}\right)\times \left(fs/800\right),$$

where fs is sampling frequency. Bandwidth $f_{b}^{i}$ of each filter can be calculated by equivalent noise bandwidth:

(9) $$f_{b}^{i}=\frac{\sum_{j}W_{ij}^{2}}{{\left(\max_{j}W_{ij}\right)}^{2}}\times \left(fs/800\right),1\leq j\leq 800.$$

**Figure 8:**
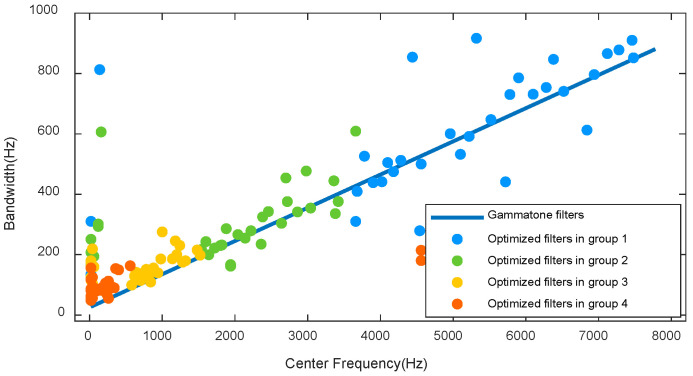
The center frequency-bandwidth distribution of optimized Gammatone kernels is plotted together with Gammatone filters.

Figure 8 shows a scatter-plot of the bandwidths against center frequencies for Gammatone filters and for optimized Gammatone filters. The optimized kernels showed a range of bandwidths at any given frequency. Frequencies of optimized Gammatone kernels did not have exact linear correlation with bandwidths compared to conventional Gammatone filters. Nonlinearities were located at low frequencies. The energy of ship radiated noise is mainly concentrated below 1 kHz. Differences in the dominant frequency of radiated noise were related to ship type [34]. The causes of the distinct spectral characteristics are unknown, but it could be reflected on learned filters to extract the differences of ship type.

#### 5.3.2. Feature Visualization and Cluster Analysis

**Figure 9:**
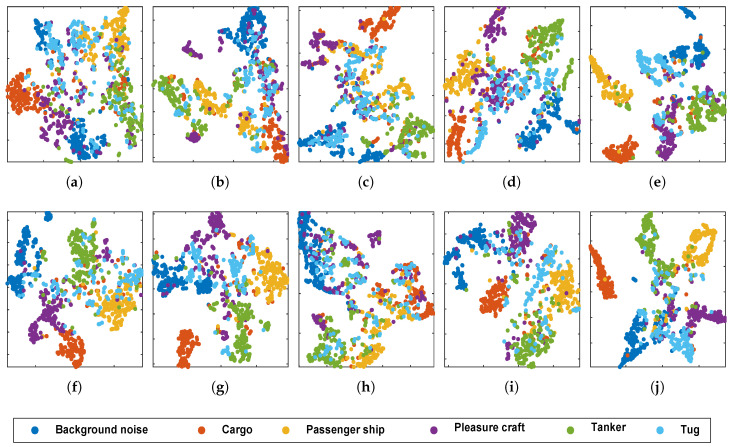
t-SNE feature visualization for features learned from proposed model and compared models. (**a**) waveform features; (**b**) wavelet features; (**c**) MFCC; (**d**) mel-frequency; (**e**) nonlinear auditory features; (**f**) spectral; (**g**) cepstral; (**h**) untrainable Gammatone initialed; (**i**) randomly initialed; (**j**) proposed.

Feature visualization method t-distributed stochastic neighbor embedding (t-SNE) [35] was used to observe features. One-thousand samples selected randomly from test datasets were used to perform the experiments. Outputs of the last full connected layer were extracted as learned features. The results are shown in Figure 9. Features in the proposed model constructed a map in which most classes were separated from other classes, except for tugs. This result was matched by the previous classification results. In contrast, there were large overlaps between many classes for features learned from hand designed features. The results indicated that features in the proposed model provided better insight into the class structure of the ocean ambient noise data.

## 6. Conclusions

In this work, we proposed an auditory inspired convolutional neural network for ship radiated noise recognition on raw time domain waveform in an end-to-end manner. The convolutional kernels in a time convolutional layer are initialized by cochlea inspired auditory filters. The choice of auditory filter banks biases our model to decompose signal into frequency components and reveal the intrinsic information of targets. Correlation statistics of signal components are analyzed by constructing a multi-scale time convolutional layer. The auditory filters are optimized in terms of ship radiated noise recognition tasks. Signal components are converted to a frequency domain by permute layer and energy pooling layer to form the “frequency map” in an auditory cortex. The whole model is discriminative trained to optimize auditory filters and deep features by objective function of ship classification.

The experimental results show that, during the training of a convolutional neural network, filter banks are adaptive in shape to improve the classification accuracy. The optimization of the auditory filter banks shape is reflected in the relationship between center frequencies and bandwidths. The proposed approach can yield better recognition performance when compared to conventional ship radiated noise recognition approaches.

Our studies developed a robust ship detection and classification model by the fusion of ship traffic data and underwater acoustic measurement. This study facilitates the development of a unique platform which could monitor underwater noise in chosen ocean areas and has automatic detection and classification capability to identify the contribution of different sources in real time.

## Figures and Tables

**Table 1:** Structure of the proposed model and compared models.

CNNs Trained on Hand Designed Features	CNNs with Same Structure as Proposed Model	Proposed Model
Extracted features: waveform, wavelet, MFCC, Mel-frequency, nonlinear auditory filter, spectral and cepstral.	1 multi-scale time convolutional layer with 128 kernels initialed randomly or initialed with untrainable Gammatone	1 multi-scale time convolutional layer with 128 kernels initialed with Gammatone
	1 permute layer	
	1 energy pooling layer	
3 convolutional layers with 32 kernels for each layer		
3 full connected layers with 32 units for each layer		
1 target layer with 6 units		

**Table 2:** Hyper-parameters of the proposed model.

Parameters	Values
Learning rate	0.0001
Batchsize	50
Epochs	84
Optimizer	RMSprop

**Table 3:** Classification results of proposed model and compared models.

Input	Features/Methods	Input Dimension	Convolutional Kernel Width	Accuracy
Hand designed features	Waveform [1,2]	8×12	5	0.574
	Wavelet [3]	14×12	5	0.679
	MFCC [8]	12×12	5	0.576
	Mel-frequency	40×12	5	0.685
	Nonlinear auditory	128×12	5	0.726
	Spectral [17,18]	2048×12	100	0.732
	Cepstral [5,6]	512×12	50	0.712
Raw time domain data	Untrainable Gammatone	4096×12	[100,200,400,800]	0.608
	Randomly initialed	4096×12	[100,200,400,800]	0.753
	Proposed model	4096×12	[100,200,400,800]	0.792

**Table 4:** Classification results of proposed method for each category.

Class	Precision	Recall	f1-Score
Background noise	0.73	0.94	0.82
Cargo	0.96	0.79	0.87
Passenger ship	0.82	0.91	0.86
Pleasure craft	0.82	0.72	0.77
Tanker	0.73	0.86	0.79
Tug	0.72	0.54	0.62
